# Purinergic P2X7 Receptor Antagonism Impairs Aerobic Capacity without Detectable Effect on Cardiac Adaptation to Exercise in Mice

**DOI:** 10.1021/acsptsci.6c00422

**Published:** 2026-09-08

**Authors:** Naiara de Souza Santos, Izabel Feltrin Fabro, Ana Paula Pinto de Araújo, Beatriz Carminati dos Santos, Tatyana Nery, Sávio de Almeida Ramos, Izabely Guedes da Rosa, Francielly Andressa Felipetti, Amanda Leite Bastos-Pereira, Aderbal S. Aguiar

**Affiliations:** † Laboratory of Exercise Biology (LABIOEX), Department of Physical Therapy, 28117 Federal University of Santa Catarina (UFSC), Araranguá, Santa Catarina 88905-120, Brazil; ‡ Laboratory of Intrauterine and Fetoplacental Analysis (LAIF), Department of Medical Sciences, Federal University of Santa Catarina (UFSC), Araranguá, Santa Catarina 88906-072, Brazil

**Keywords:** P2X7 receptor, brilliant blue G, aerobic capacity, exercise training, cardiac remodeling, purinergic signaling

## Abstract

Adenosine triphosphate (ATP) signaling through the P2X7 receptor is an emerging determinant of mitochondrial energy metabolism and physical fitness, and the receptor is increasingly targeted pharmacologically for its role in inflammation. Whether systemic P2X7 antagonism affects aerobic capacity and the structural adaptation of the heart to exercise is unknown. Adult male Swiss mice were studied in a 2 × 2 factorial design crossing physical conditioning (sedentary or exercised) with treatment (saline or the P2X7 antagonist Brilliant Blue G, BBG; 50 mg/kg). Exercised animals trained on a treadmill on alternate days over approximately 4 weeks; maximal oxygen uptake (V̇O_2_max) and power were measured by incremental treadmill ergospirometry, and myocardial cardiomyocyte volume density (Vv) was quantified by design-based stereology. Aerobic training increased V̇O_2_max and cardiomyocyte Vv. BBG reduced V̇O_2_max and peak power independently of training and produced a deficit that widened progressively with workload across the incremental test, indicating a demand-dependent limitation of oxidative performance. Critically, BBG did not detectably alter the exercise-induced increase in cardiomyocyte Vv and showed no interaction with training; motor coordination, locomotor and exploratory behavior, blood lactate, and body mass were also unaffected. P2X7 antagonism thus impairs aerobic capacity without detectable blunting of the structural response of the heart to exercise. This functional–structural dissociation points to a metabolic process, plausibly mitochondrial, rather than to cardiac remodeling, although the present sample size bounds rather than excludes an effect on cardiac adaptation, and indicates that P2X7-directed therapies may reduce exercise capacity without producing overt cardiac structural change.

## Graphical abstract

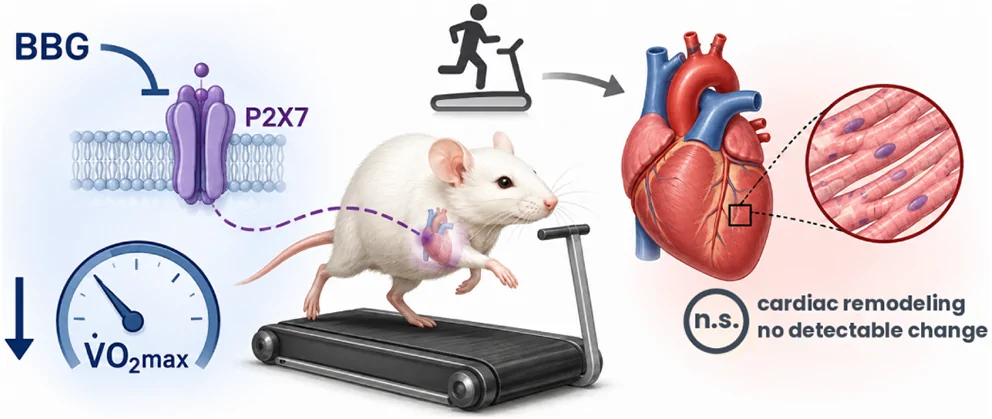

## Introduction

Adenosine triphosphate (ATP) is the principal cellular energy currency, but it also acts as an extracellular signaling molecule, activating purinergic receptors that are widely distributed across tissues. These receptors comprise the ionotropic P2X and the metabotropic P2Y families.^1^ Among the seven ionotropic subtypes, the P2X7 receptor stands out as an ATP-gated cation channel expressed at the membrane of immune cells—monocytes, macrophages, and microglia—but also in mitochondria and in cardiomyocytes.^2,3^ First identified as a cytolytic receptor,^4^ P2X7 is now recognized as a central node of inflammation, its overactivation driving assembly of the NLRP3 inflammasome and release of interleukin-1β.^2^ This pro-inflammatory role, however, coexists with a less explored bioenergetic function.

The mitochondrial localization of P2X7 suggests a direct role in the regulation of energy metabolism. Within this compartment, the receptor modulates membrane potential, calcium homeostasis, and oxidative phosphorylation, and it senses cytosolic ATP and signals through kinase-dependent pathways.^5^ P2X7 is a modulator of glucose and energy metabolism: it regulates aerobic glycolysis,^6^ and altering its activity changes whole-body energy expenditure and substrate utilization.^7^ Convergently, basal mitochondrial expression of P2X7 enhances ATP synthesis, mitochondrial respiration, and physical performance, positioning the receptor as a bioenergetic determinant of physical fitness.^3^

This metabolic function takes on particular relevance in the heart, which is an organ of high oxidative demand that is heavily dependent on oxidative phosphorylation. Purinergic signaling is integral to cardiovascular regulation at multiple levels,^8^ and P2X7 is well expressed in cardiomyocytes. In humans, a loss-of-function mutation in the P2X7 gene (E186 K) was associated with hypertrophic cardiomyopathy in a family with a history of sudden death, implicating the receptor in heart failure mediated by arrhythmias.^9^ In animal models, hearts of P2X7-deleted mice are larger, contain smaller mitochondria, and show reduced stroke volume, ejection fraction, fractional shortening, and cardiac output.^3^ Together, these findings indicate that P2X7 contributes to the maintenance of the normal cardiac structure and function.

P2X7 can be pharmacologically antagonized by Brilliant Blue G (BBG), a noncompetitive antagonist whose site of action has been mapped by mutagenesis to the intersubunit allosteric pocket of the receptor ectodomain.^10–12^ Whether systemically administered BBG reaches the central nervous system remains unsettled. After spinal cord injury in rats, BBG accumulates in the contused cord but is minimal in tissue only millimeters away, which the authors attribute to entry through the disrupted blood–spinal cord barrier;^13^ in intact rodents, brain exposure after an equivalent dose is roughly two orders of magnitude below that in plasma and is not accompanied by detectable brain P2X7 occupancy.^14^ BBG was selected for two reasons. It is the compound through which the in vivo pharmacology of P2X7 has largely been established, including the metabolic and physical-performance phenotypes against which the present findings must be read,^3,6,7,15^ and it has been administered repeatedly in mice over protocols longer than the one used here—50 mg·kg^–1^ intraperitoneally three times weekly for up to 10 weeks and 50–75 mg·kg^–1^ twice weekly for 4 weeks—producing anti-inflammatory and tissue-protective effects consistent with receptor blockade, and without adverse effects attributable to the compound in either model.^16,17^ BBG is not a clean probe, however: it inhibits neuronal voltage-gated sodium channels (IC_50_ ≈ 2 μM),^18^ blocks pannexin-1 channels and pannexin-1-mediated ATP release at 1 μM,^19^ and inhibits the rat P2X2 receptor with an IC_50_ of approximately 1.4 μM^10^—concentrations of the same order as the total plasma exposure reached at this dose, though the free fraction is lower because BBG binds proteins avidly.^13^ The present design, therefore, includes motor and locomotor readouts that bear directly on these liabilities, and we return to them in the Discussion.

Chronic aerobic exercise is a potent adaptive stimulus for both oxidative metabolism and cardiac structure. Endurance training promotes physiological cardiac hypertrophy—characterized by sarcomere addition, metabolic and mitochondrial adaptation, and preserved or enhanced contractile function—distinct from pathological remodeling.^20–22^ In parallel, it raises maximal oxygen uptake (V̇O_2_max), the gold-standard measure of aerobic capacity. In rodents, V̇O_2_max can be determined by treadmill ergospirometry coupled with maximal incremental testing^23,24^ under strict control of indirect-calorimetry conditions;^25^ the running speed reached in the incremental test further allows training to be prescribed and monitored and power to be estimated.^26–29^

Myocardial structural adaptation, in turn, can be quantified in an unbiased and reproducible manner by design-based stereology, which estimates parameters such as volume density (Vv) from two-dimensional sections.^30^ Despite evidence that P2X7 is relevant to both energy metabolism and cardiac integrity, it remains unknown how its pharmacological antagonism interacts with aerobic training on aerobic capacity and cardiac structure. Compounding this gap, much of the evidence on the role of P2X7 in physical performance rests on motor-coordination tests, such as the rotarod, rather than on direct measures of aerobic capacity.^3,31^

Given this gap, the present study evaluated the combined effects of aerobic training and P2X7 receptor antagonism with BBG in mice. Specifically, the aims were **(I)** to assess the effect of P2X7 antagonism on aerobic capacity through performance markers such as V̇O_2_max and power; **(II)** to determine the effect of P2X7 antagonism on the structural remodeling of the myocardium, testing whether BBG blunts the exercise-induced cardiac adaptation; and **(III)** to analyze motor coordination and locomotor and exploratory behavior in order to exclude nonspecific effects on performance.

## Results and Discussion

Antagonism of the P2X7 receptor with Brilliant Blue G (BBG) impaired aerobic capacity without a detectable effect on exercise-induced cardiac structural adaptation, motor performance, or exploratory behavior. Because the P2X7 receptor is a determinant of mitochondrial energy metabolism and physical fitness^3^ and because its pharmacological blockade is being pursued as an anti-inflammatory strategy, defining which components of exercise physiology depend on P2X7 activity is of direct translational relevance for the safety of P2X7-targeted therapeutics in physically active individuals. Aerobic training increased maximal oxygen uptake (V̇O_2_max) and cardiomyocyte volume density, whereas BBG produced a convergent aerobic deficit—lower V̇O_2_max, lower peak power, and a progressive worsening of performance across the incremental test—with no parallel reduction in cardiac remodeling and no change in motor coordination, locomotion, or blood lactate. This dissociation between a measurable aerobic deficit and a cardiac structural adaptation on which no effect was detected is the central finding of this study and points to a metabolic process, plausibly mitochondrial, rather than to cardiac remodeling or motor function (Figure 1).

**Figure 1:**
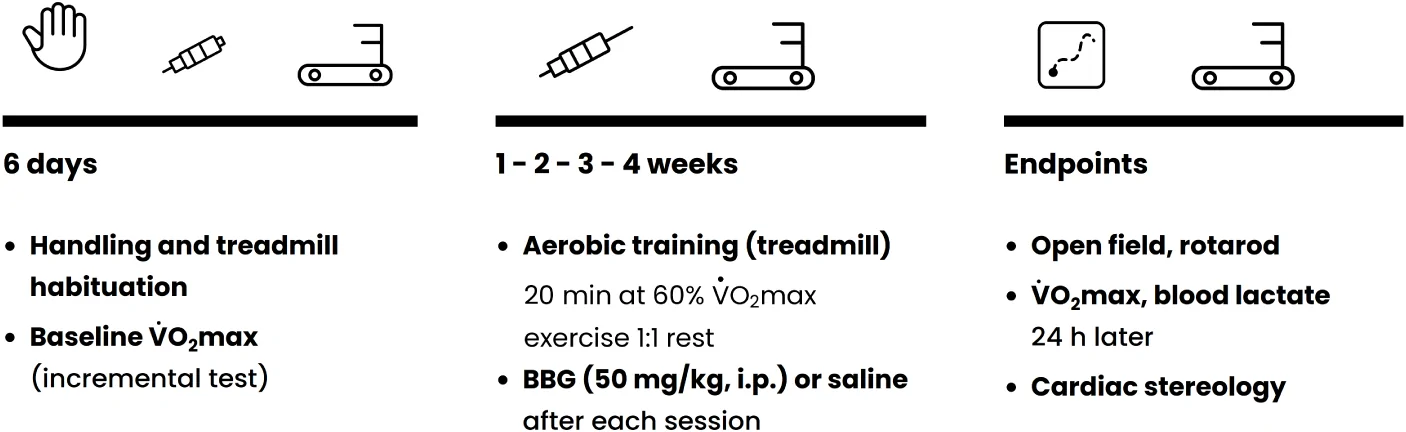
Experimental design. Adult male Swiss mice were handled and habituated to the treadmill over 6 days and underwent a baseline incremental test to determine maximal oxygen uptake. Exercised groups then trained on the treadmill on alternate days, in a 1:1 exercise-to-rest ratio (16 sessions, 20 min each, at 60% of the baseline maximum), while sedentary groups were placed on the stationary treadmill on the same days but did not run. Immediately after each session and at the matched time of day for sedentary animals, every animal received an intraperitoneal injection of BBG (50 mg·kg^–1^) or an equal volume of 0.9% saline according to group allocation. Injections were also given after the open-field and rotarod tests, which were performed on consecutive days, with the rotarod 24 h before the final incremental test and blood sampling, after which hearts were collected for stereology.

Aerobic capacity was assessed by maximal incremental treadmill ergospirometry and analyzed by Type III two-way ANOVA (training × treatment) with sum-to-zero coding. Training increased absolute V̇O_2_max (main effect *F*(1,28) = 12.30, *p* = 0.0015, η*p* ^2^ = 0.31, 90% CI [0.08, 0.48]; trained vs sedentary Hedges’ *g* = 1.12 [0.44, 1.96]), a result corroborated by aligned rank transform (ART) ANOVA (*F* = 14.0, *p* = 0.001). BBG reduced V̇O_2_max independently of training status (treatment *F*(1,28) = 7.12, *p* = 0.013, η*p* ^2^ = 0.20 [0.03, 0.39]; saline vs BBG *g* = 0.79 [0.15, 1.61]; ART *F* = 8.41, *p* = 0.007), with no training × treatment interaction (*F* = 0.02, *p* = 0.90). Exercise and P2X7 blockade thus exerted opposing, additive influences on maximal aerobic capacity: training raised V̇O_2_max by roughly one standard deviation, while BBG lowered it by a comparable margin in both sedentary and trained animals (Figure 2D).

**Figure 2:**
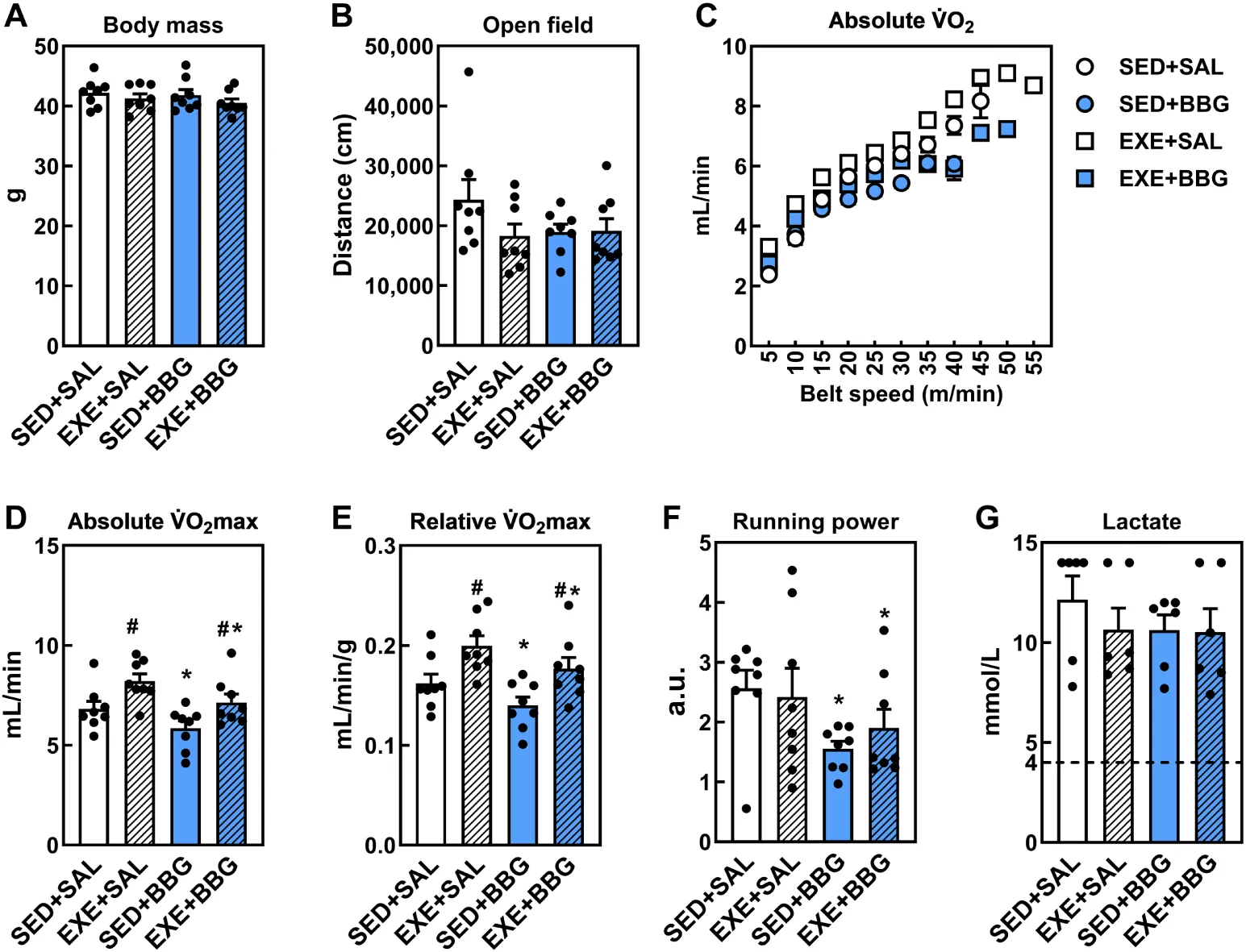
Aerobic capacity and locomotor behavior after aerobic training and P2X7 antagonism with Brilliant Blue G (BBG). (A) Body mass. (B) Total distance traveled in the open field. (C) Stagewise absolute oxygen uptake (V̇O_2_) across the incremental treadmill test. (D) Absolute maximal oxygen uptake (V̇O_2_max). (E) Mass-normalized (relative) V̇O_2_max. (F) Running power. (G) Blood lactate after the maximal test (dashed line, 4 mmol·L^–1^). Groups: sedentary–saline (SED+SAL), exercised–saline (EXE+SAL), sedentary–BBG (SED+BBG), and exercised–BBG (EXE+BBG). Open bars, saline; blue bars, BBG; hatching denotes trained groups. Bars are mean ± SEM with individual values (n = 8 per group, except blood lactate, n = 6 per group). #, main effect of training; *, main effect of BBG (two-way ANOVA, Type III sums of squares; α = 0.05). Within the aerobic family, the BBG effects on V̇O_2_max and running power are convergent under false-discovery-rate control but do not survive family-wise (Holm) correction (see Methods).

The same pattern was evident for peak power output (Figure 2F), which was reduced by BBG (*F*(1,28) = 5.43, *p* = 0.027, η*p^2^* = 0.16; *g* = 0.82) but unaffected by training (*p* = 0.76); the absence of a training effect on power is consistent with the limited dynamic range of the power estimate at the running speeds used. We treat these aerobic end points conservatively. Within the predefined family of correlated aerobic outcomes, the BBG effects on V̇O_2_max and peak power lie at the Benjamini–Hochberg false-discovery-rate boundary (adjusted *p* ≈ 0.05) but do not survive the more stringent Holm family-wise correction, and the effect on mass-normalized V̇O_2_max (Figure 2E) is the least robust of the set (ART *p* = 0.051, with a bootstrap interval touching zero). The defensible inference is, therefore, not that any single end point independently withstands family-wise control, but that several correlated, prespecified measures of aerobic function shift coherently in the same direction under P2X7 blockade.

The aerobic deficit was demand-dependent. Modeling the stagewise V̇O_2_ trajectory of the incremental test (Figure 2C) with a linear mixed-effects model (random intercept per animal, stage as a categorical factor) revealed a significant stage × treatment interaction (parametric bootstrap likelihood-ratio test, *p* = 0.002): BBG-treated animals diverged progressively from saline controls as the workload increased, rather than differing by a fixed offset. A deficit that widens with metabolic demand points to a ceiling on oxidative ATP supply rather than to a difference in resting metabolism. This is consistent with the mitochondrial localization of the P2X7 receptor and its role as a bioenergetic sensor supporting oxidative phosphorylation and ATP synthesis:^3,5^ genetic loss of P2X7 function lowers whole-body energy expenditure and fatty-acid oxidation^15^ and depresses mitochondrial respiration,^3^ and, in cells engineered to express it, the receptor upregulates glucose transport and glycolytic enzymes.^6^ How a membrane-impermeant antagonist produces such a phenotype requires comment. BBG acts from the extracellular face of the receptor—mutation of residues lining the intersubunit allosteric pocket at the apex of the ectodomain (D92A, F103A) essentially abolishes its antagonist activity^12^—and there is no evidence that it crosses the plasma membrane. The pool it can engage is, therefore, the plasma-membrane pool, and the mitochondrial component of the phenotype must be a downstream consequence of that blockade rather than a direct action on mitochondrial receptors. This is less a limitation of the proposed mechanism than a specification of it. P2X7 has an unusually low affinity for ATP, and interstitial ATP rises severalfold in contracting skeletal muscle; the receptor has accordingly been proposed to act as a bioenergetic sensor that engages the intracellular ATP-synthetic machinery only when extracellular ATP accumulates, that is, when energy demand is high.^3^ Blockade of surface P2X7 would, therefore, be expected to withdraw metabolic support precisely under maximal demand and to be silent at rest, which is the pattern observed here. We note that the mitochondrial phenotype of P2X7-deficient cells has been attributed principally to reduced Complex I activity, with lower matrix Ca^2+^ a consequence rather than the cause,^3^ and that how a blockade restricted to the cell surface reproduces that intracellular phenotype is not established. The reciprocal manipulation supports this reading. Sustained stimulation of P2X7 in healthy mice raises oxygen consumption and energy expenditure and upregulates NADPH oxidase 2 in both mixed and fast-twitch skeletal muscle, a profile its authors describe as resembling the adaptations produced by endurance training.^7^ The present study performs the converse manipulation, over a longer period and in an exercise paradigm, and obtains the converse outcome.

In contrast to its functional effect, BBG did not detectably alter the structural adaptation of the heart to training. Stereological point counting showed that exercise increased the volume density of cardiomyocytes in the myocardium (Figure 3A,D–G; Vv; main effect of training, *F*(1,21) = 9.29, *p* = 0.006, η*p* ^2^ = 0.31), an adaptation consistent with physiological cardiac remodeling, in which myocyte growth predominates over fibrosis and contractile function is preserved or enhanced.^22,32^ The extracellular-matrix compartment (Figure 3B), by contrast, was unchanged by either training or treatment (all *p* > 0.27), indicating that the increased cardiomyocyte volume density reflected myocyte growth rather than interstitial fibrosis. Critically, BBG produced no main effect on cardiomyocyte Vv and no training × treatment interaction, indicating that P2X7 blockade neither mimicked nor blunted the exercise-induced increase in myocardial cellular density; the adaptation was a change in volume fraction without a detectable change in absolute heart volume estimated by fluid displacement (Figure 3C). Bounding these null effects, the 90% confidence interval for the main effect of BBG on cardiomyocyte Vv was −0.034 to +0.023 and that for the training × treatment interaction was −0.090 to +0.024, against a training effect of +0.051 (90% CI, +0.022 to +0.080) estimated in the same model. The design, therefore, excludes a main effect of BBG larger than about two thirds of the training effect, but does not constrain the interaction: the exercise-induced increase in cardiomyocyte Vv remained present under BBG (+0.034), yet an attenuation of that increase cannot be excluded. This is the crux of the dissociation, and also its limit: the treatment that measurably impaired aerobic function produced no detectable change in the cardiac structural response to training, at a resolution that bounds rather than excludes such a change. The comparison with genetic models is instructive. Constitutive deletion of P2X7 in mice produces enlarged hearts with smaller mitochondria and reduced stroke volume, ejection fraction, and cardiac output alongside its metabolic deficits;^3^ here, acute pharmacological antagonism reproduced the functional and metabolic deficit without a detectable counterpart in the structural readout used, although that readout does not cover the contractile end points altered in the null mice. The most parsimonious interpretation is that the cardiac remodeling defect of P2X7-null mice reflects chronic, possibly developmental, loss of the receptor, whereas short-term blockade in the adult constrains oxidative performance without a detectable effect on the signaling that drives physiological hypertrophy—favoring a metabolic and mitochondrial locus for the effect of BBG, without excluding a structural component below the resolution of the present design.

**Figure 3:**
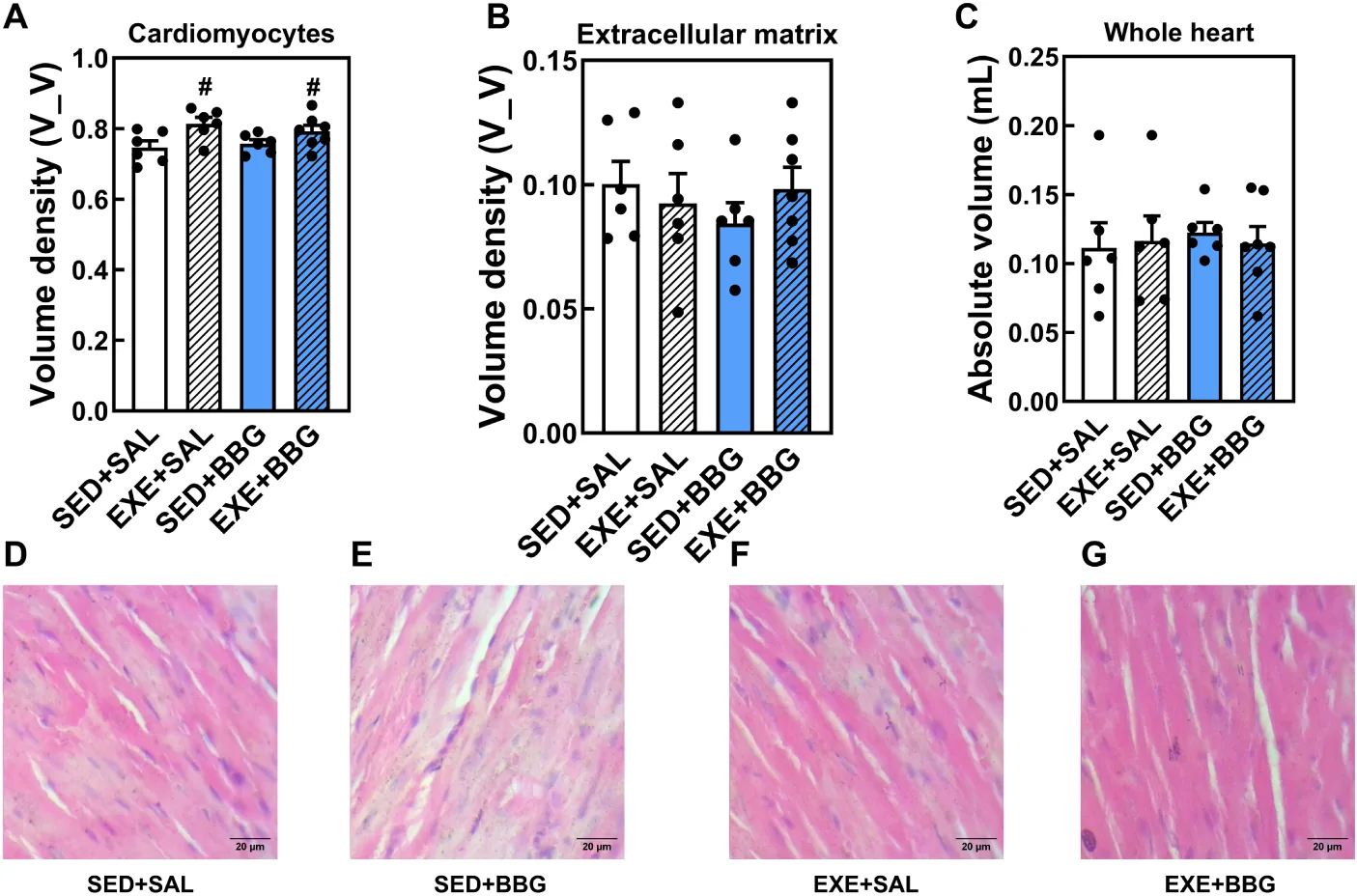
Cardiac structure after aerobic training and P2X7 antagonism with Brilliant Blue G (BBG). (A) Volume density (Vv) of cardiomyocytes. (B) Volume density of the extracellular matrix. (C) Absolute heart volume estimated by fluid displacement. (D–G) Representative photomicrographs of the myocardium stained with hematoxylin and eosin: SED+SAL (D), SED+BBG (E), EXE+SAL (F), and EXE+BBG (G); scale bar, 20 μm. Bars are mean ± SEM with individual values (n = 6–7 per group). #, main effect of training (two-way ANOVA, Type III sums of squares; α = 0.05); no effect of BBG or training × treatment interaction was detected for any cardiac end point.

The aerobic deficit cannot be attributed to nonspecific effects of BBG. Locomotor and exploratory activity in the open field (Figure 2B) were unaffected by training or treatment (all *p* > 0.18; ART confirming), excluding a generalized reduction in movement or motivation. Blood lactate measured immediately after the maximal test (Figure 2G) did not differ across groups (treatment *p* = 0.44; training *p* = 0.46), indicating that all animals reached a comparable degree of metabolic strain at exhaustion and that the test elicited maximal effort irrespective of treatment. Body mass (Figure 2A) was also unaffected (*p* > 0.18), ruling out body-composition differences as an explanation for the divergence in V̇O_2_max. Motor coordination, assessed by the rotarod, was likewise unaffected: neither training (*F*(1,20) = 0.80, *p* = 0.38) nor BBG (*F*(1,20) = 0.16, *p* = 0.70) altered the latency to fall, and no interaction was detected (*F*(1,20) = 2.72, *p* = 0.11), with the aligned rank transform confirming the absence of effects (all *p* ≥ 0.12). Preserved rotarod performance indicates that the aerobic deficit does not stem from gross motor or coordination impairment, reinforcing a metabolic rather than neuromuscular locus for the effect of BBG. These same controls bear on the off-target pharmacology of BBG. Peak plasma exposure in rodents at this dose is approximately 2 μM,^14^ of the same order as the concentrations at which BBG inhibits voltage-gated sodium channels^18^ and pannexin-1 channels,^19^ although the free fraction is lower than this total because BBG binds proteins avidly.^13^ Inhibition of voltage-gated sodium channels by BBG is strongly state-dependent: IC_50_ falls from 171 μM at −120 mV to approximately 10 μM at −80 mV and 2 μM at −60 mV, reflecting high-affinity binding to inactivated states, and the block accumulates with repetitive stimulation.^18^ At the resting potentials of nerve and muscle, the block expected at the present exposure is, therefore, modest, but it would accumulate during sustained high-frequency firing. The rotarod and the open field impose no such demand, and we do not claim that their null results exclude a use-dependent sodium-channel contribution to the aerobic deficit. Central engagement is less likely still since brain exposure at this dose is approximately 15–26 nM, some two orders of magnitude below the sodium-channel IC_50_.^14^ Pannexin-1 blockade cannot be excluded, but pannexin-1 is the ATP-release channel that gates P2X7, so its inhibition would relocate the effect within purinergic signaling rather than outside it. What supports a receptor-level interpretation is the convergence of the present result with three orthogonal manipulations of P2X7: constitutive deletion depresses mitochondrial respiration and shortens the latency to fall on the rotarod,^3^ loss of receptor function lowers whole-body energy expenditure and fatty-acid oxidation,^15^ and receptor stimulation raises whole-body energy expenditure and oxygen consumption.^7^ Neither sodium-channel nor pannexin-1 blockade offers a reason to reproduce the direction of a P2X7 deletion. The genetic and pharmacological phenotypes differ in extent, as would be expected. Constitutive deletion is lifelong and complete and is accompanied by larger hearts, reduced stroke volume and ejection fraction, and a lower resting body temperature;^3^ 4 weeks of antagonism produced no enlargement of the heart, although stroke volume, ejection fraction, cardiac output, and body temperature were not measured here, and the rotarod deficit reported in those animals is most plausibly a consequence of that systemic phenotype rather than of a motor requirement of the receptor. A narrower phenotype under pharmacological blockade than under constitutive deletion is the expected relationship, and it is seen with a selective antagonist too, which lowers energy expenditure less than gene deletion does.^15^ This dissociation also runs in the opposite direction: stimulation of P2X7 raises whole-body energy expenditure and oxygen consumption in mice without altering spontaneous locomotor activity,^7^ as blockade here reduced maximal oxygen uptake without altering it. A metabolic action of P2X7 that leaves locomotor readouts unchanged is therefore the expected pattern rather than an unexplained one.

A further question is where within the periphery the constraint acts. P2X7 receptors are present on presynaptic motor nerve terminals of mouse skeletal muscle, though not on terminal Schwann cells or postsynaptic fibers,^33,34^ and functional P2X7 responses have also been described in cultured Schwann cells.^35^ At the motor terminal, P2X7 activation promotes vesicle release, and 1 μM BBG—the compound and approximately the concentration relevant here—abolishes that release through the receptor’s ion channel rather than its macropore;^34^ P2X7-dependent enhancement of neuromuscular transmission has since been characterized further.^36^ A graded reduction in transmission efficiency would not necessarily produce deficits in spontaneous locomotion or rotarod performance, both of which are brief and impose modest demands on sustained firing, yet could curb motor output during an incremental test, and would do so progressively with workload as motor-unit firing rates rise. Moreover, although ecto-ATPases are thought to keep synaptic ATP below the threshold for P2X7 activation at rest, that threshold falls when extracellular divalent cations decline and ATP is released from both nerve terminals and contracting fibers^34^—conditions approached during maximal exercise. We therefore cannot separate a neuromuscular from a metabolic route on the present data. Two observations bear on the balance between them. Blood lactate at exhaustion did not differ between treatments, which is not what a purely neuromuscular ceiling would predict, since animals limited by transmission failure would be expected to stop short of comparable metabolic strain. And in a mouse model of amyotrophic lateral sclerosis, stimulation of P2X7 restored the glycolytic fiber composition of the tibialis anterior and improved mitochondrial coupled respiration,^37^ so blockade at the muscle would be expected to act in the same direction as the metabolic account. That study bears on the neuromuscular route as well, and in the opposite sense: the same treatment preserved endplate morphology and reduced denervation.^37^ A receptor whose activation is trophic at the junction is one whose blockade could plausibly degrade transmission there, so this evidence strengthens rather than resolves the alternative raised above. Both routes are P2X7-mediated and are not mutually exclusive; the defensible statement is that P2X7 blockade constrains aerobic performance at a peripheral locus that the present design does not resolve further.

One asymmetry should be noted when reading the present pharmacological data alongside the genetic and pharmacological evidence to which they converge. Those studies were performed in C57BL/6 mice,^3,7,15^ a line fixed for the hypofunctional 451L allele; the authors who characterized that allele caution that P2X7 phenotypes measured against such backgrounds may be underestimated.^38^ The direction of those effects is unaffected by this, and it is the direction rather than magnitude on which the present comparison rests, but effect sizes across these designs are not directly comparable.

Several limitations qualify these conclusions. A single dose and post-session timing of BBG were used, so dose–response relationships and the kinetics of the aerobic deficit remain undefined. Systemic administration does not separate peripheral from central P2X7 blockade, so the relative contributions of cardiac, skeletal-muscle, and neural P2X7 to the aerobic phenotype cannot be resolved here. The available evidence, nonetheless, favors a peripheral locus. The central accumulation of BBG reported after spinal cord injury tracked the lesioned tissue and was attributed by those authors to a disrupted blood–spinal cord barrier,^13^ and in intact rodents, brain exposure is low and unaccompanied by detectable brain P2X7 occupancy.^14^ We note that neither study measured brain exposure in an animal with an intact barrier after intraperitoneal dosing and that whether the repeated dosing used here permits central accumulation has not been tested.^14^ BBG-treated animals developed visible blue discoloration of the skin during the protocol. Coloration of cardiac or brain tissue was not recorded at necropsy, and we did not undertake further animal experiments to document it since the anatomical distribution of BBG in rodents has already been quantified.^13,14^ The structural readout was a stereological volume density; absolute cardiomyocyte volume and number were not estimated, and no change in absolute heart volume was detected, so hypertrophic or hyperplastic responses below the resolution of displacement volumetry cannot be excluded. The cardiac arm of this dissociation is a null result in six to seven animals per group and is not powered to exclude a biologically meaningful effect. The training-induced increase in cardiomyocyte Vv was +0.067 under saline and +0.034 under BBG; the training × treatment interaction was not significant (p = 0.33), and the difference between a significant and a non-significant simple effect is not itself evidence of a difference, but the confidence interval for that interaction spans attenuations ranging from complete abolition of the training response to a modest enhancement. Detecting an attenuation of half the training effect would require approximately 85 animals per group at 80% power. The absence of a detectable effect on cardiac remodeling should, therefore, be read as a limit on what the present design can resolve rather than as evidence that cardiac adaptation is unaffected: the dissociation reported here is between a measurable functional deficit and a structural response that this study could not resolve. Finally, the mitochondrial mechanism we infer was not measured directly—respirometry, ATP flux, and oxidative-phosphorylation readouts would be required to confirm that the demand-dependent deficit reflects constrained mitochondrial oxidative capacity rather than an upstream limitation in oxygen delivery.

A further limitation concerns the subcellular site of blockade. There is no evidence that BBG penetrates cells and none that it engages the mitochondrial pool of P2X7; we, therefore, attribute the present effect to blockade of plasma-membrane receptors and treat the mitochondrial component as inferred rather than demonstrated. This limitation is not peculiar to the pharmacology used here: constitutive deletion of *P2rx7* removes the plasma-membrane and mitochondrial pools together,^3^ so the available genetic evidence does not apportion the metabolic phenotype between them either. Resolving the contribution of mitochondrial P2X7 will require compartment-selective tools rather than systemic antagonism. A related limitation concerns the probe itself. BBG remains the most widely used compound for interrogating P2X7 in vivo, but its selectivity has been questioned, and structurally unrelated antagonists have been recommended in its place for establishing receptor-driven mechanisms.^14^ The present design cannot substitute for that comparison, and that comparison is practicable: a selective antagonist has been given peripherally and repeatedly in mice, A-804598 at 90 mg·kg^–1^ intraperitoneally for seven consecutive days.^15^ Replication with a selective antagonist such as A-804598 or JNJ-47965567, and with *P2rx7*-null mice, is the necessary next step and would convert the present pharmacological inference into a receptor-level one. A species caveat attaches to the selectivity itself. The margin of BBG for P2X7 over the other P2X subtypes was established in rats, where it exceeds three orders of magnitude; at the human receptor, the potency is roughly 25-fold lower and the margin over P2X4 narrows to about 15-fold.^10^ The mouse receptor has not been characterized in this respect, so the selectivity window that applies to the present experiments is not known.

Two features of the animal model further qualify the interpretation. The mouse *P2rx7* gene carries a natural P451L polymorphism in the cytoplasmic tail that impairs receptor function; of 14 strains surveyed, only C57BL/6, C57BL/10, DBA/1, and DBA/2 carried the 451L allele, every other strain—including four derived from wild mice—carrying 451P, which those authors take to be the wild type within the mouse population.^38^ Swiss was not among the strains tested; we did not genotype our animals, and because the stock is outbred, its allele distribution cannot be assumed. Two considerations bound the consequences. The substitution lies in the intracellular C-terminus, whereas BBG binds the receptor ectodomain,^12^ so the polymorphism cannot alter the antagonist binding site. And any carriage of 451L would reduce baseline receptor function and, therefore, attenuate rather than inflate the effect of an antagonist, as would the greater within-group genetic variance of an outbred stock; the present estimates are conservative in both respects. A study performed in C57BL/6 would have carried the opposite risk since that line is fixed for the hypofunctional allele, and the authors who identified it note that P2X7 phenotypes measured on such backgrounds may have been underestimated.^38^ Only males were studied, so these findings do not establish whether P2X7 blockade affects aerobic capacity in females. Extending the design would not be a matter of adding a sex factor: estrous-cycle phase itself modulates submaximal oxygen uptake and exercise thermoregulation,^28^ so a female arm would require cycle staging to avoid confounding the very end points at issue.

None of these qualifications bears on the principal observation. Aerobic capacity was impaired, and no effect on cardiac structural adaptation was detected in the same animals under the same treatment; what remains open is the tissue and the process through which P2X7 blockade imposes that limit and the magnitude of any cardiac effect the present sample could not resolve.

In summary, pharmacological antagonism of the P2X7 receptor with BBG impairs aerobic capacity in a manner that intensifies with metabolic demand, yet without a detectable effect on the structural adaptation of the heart to aerobic training. Read alongside the impaired performance of P2X7-deficient mice, this functional–structural dissociation supports P2X7 as a bioenergetic determinant of maximal aerobic performance whose acute blockade is limiting metabolically, with no structural counterpart detectable at the present sample size, and it cautions that P2X7-directed anti-inflammatory therapies could blunt exercise capacity without producing overt cardiac structural change—a phenotype that structural end points alone would miss.

## Methods

### Animals and Ethics

Adult male Swiss mice (8–10 weeks; 25–45 g) from the UFSC Central Animal Facility were studied. Males were used because the primary end points of this design are stagewise oxygen uptake and running power at fixed belt speeds, and the estrous cycle acts on those measurements directly: proestrus raises submaximal oxygen uptake and carbon dioxide production at low running speeds, and estrus impairs exercise-induced thermoregulation.^28^ We note that those authors conclude that the estrous cycle should not by itself exclude females from exercise designs; the constraint here is narrower—a female arm would have required estrous-cycle staging rather than the simple addition of a sex factor (see Discussion). The outbred Swiss stock, maintained by the institutional facility, was chosen in preference to an inbred line because it is not fixed for any allele at the *P2rx7* locus, including the hypofunctional P451L variant, which among 14 strains surveyed was confined to the C57BL and DBA lines;^38^ animals were not genotyped at this locus (see Discussion). Mice were housed in collective cages under a controlled 12 h light/dark cycle at 22 ± 2 °C, with environmental enrichment and free access to water and chow. All procedures complied with the Brazilian guidelines for animal experimentation (Law 11,794/2008 and CONCEA regulations) and were approved by the Ethics Committee on Animal Use of the Federal University of Santa Catarina (CEUA/UFSC; protocol no. 6921230724). The ergospirometric and open-field end points were analyzed in 32 animals (n = 8 per group), the rotarod in 24 animals (n = 6 per group), and cardiac stereology in 25 animals (n = 6–7 per group).

Experimental design and handling. Animals were assigned to a 2 × 2 factorial design, crossing physical conditioning (sedentary or exercised) with treatment (0.9% saline or BBG). Over the 3 days preceding the protocol, all animals were handled for 5 min/day, including a simulated intraperitoneal injection and 5 min on the switched-off treadmill, and were rewarded with a sunflower seed to minimize handling stress.^39,40^

### Incremental Maximal Test and Ergospirometry

Maximal aerobic capacity was assessed by an incremental treadmill test with indirect calorimetry at the beginning and end of the protocol, preceded by a three-day habituation (5 min on the stationary treadmill with a 0.2 mA stimulus applied only when the animal rested on the rear grid, followed by running at 5, 10, and 15 m·min^–1^ for 600 s on consecutive days after a 120 s warm-up at 5 m·min^–1^). During the test, speed was increased by 5 m·min^–1^ every 120 s until exhaustion, defined as three consecutive contacts with the shock grid within 10 s. Gas supply (≈21% O_2_, ≈0.03% CO_2_) was maintained at 1200 mL·min^–1^ with the treadmill inclined at 1.7°. Oxygen uptake was recorded throughout by indirect calorimetry with a gas analyzer (oxygen sensor, LOX–O2-F; Bonther, Brazil) coupled to a metabolic treadmill (EB 500; Bonther, Brazil); V̇O_2_max was taken as the highest V̇O_2_ attained, and peak and stagewise power were estimated from body mass and running speed as described previously.^28,29^

### Training Protocol

Exercised animals ran on the treadmill on alternate days, in a 1:1 exercise-to-rest ratio, completing 16 sessions of 20 min, at the speed corresponding to 60% of the V̇O_2_max determined in the baseline incremental test; this speed was set from that test and was not readjusted. Sedentary animals were placed on the stationary treadmill on the same days and removed after an equivalent interval so that all animals followed the same experimental sequence but did not run. Immediately after each session, every animal received one intraperitoneal injection of BBG (50 mg·kg^–1^) or an equal volume of 0.9% saline according to group allocation. Injections were also given after the open-field and rotarod tests so that each animal received 18 injections in total; no injection was given on the day of either incremental test. The full schedule is shown in Figure 1.

### Blood Lactate

After 24 h of rest, the animals repeated the incremental test, and blood was collected from the submandibular vein. Lactate was measured immediately with reagent strips (Lactate Detect Strip, BS.0002) and a portable analyzer (Lactate Detect/TD-4261) as recommended by the manufacturer. Lactate could not be determined in two animals per group because of reagent-strip failure and reader error, so this end point has n = 6 per group.

### Open Field

Spontaneous locomotion and exploratory behavior were assessed in an open-field arena (90 cm diameter; EP 154G, Insight, Brazil).^41^ Each animal was placed in the center and allowed 5 min of free exploration without prior habituation. Sessions were recorded and analyzed with ANY-maze software (Stoelting Co., USA), quantifying total distance traveled, time in the center and in the periphery, and central distance.

### Rotarod

Motor coordination and balance were assessed on a rotarod.^42^ Animals were habituated at 0 rpm for 30 s and at 5 rpm for 90 s and returned to their cages; after 30 min, the test was run for 5 min with rotation accelerating up to 30 rpm, recording the latency to fall and the time on the rod.

### Euthanasia and Cardiac Stereology

At the end of the protocol, animals were euthanized by an anesthetic overdose (ketamine, 300 mg·kg^–1^, plus xylazine, 30 mg·kg^–1^, intraperitoneally), and the hearts were excised, fixed by immersion in 4% formaldehyde, and weighed. Total cardiac volume was estimated by fluid displacement (Scherle’s principle), recording the primary (fresh) volume, the post-fixation volume, and the absolute total volume corrected for 10% tissue shrinkage. Hearts were embedded, sectioned at 5 μm, and stained with hematoxylin and eosin for design-based stereology.^30^ The volume densities (Vv) of the cardiomyocyte and extracellular-matrix compartments of the myocardium were estimated by point counting with a 144-point test grid applied to seven systematically sampled microscopic fields per animal under a light microscope (NO226T3; Global Optics, Brazil) with a 40× objective (numerical aperture 0.65), Vv being calculated as the mean fraction of test points falling on each compartment.

### Statistical Analysis

Analyses were performed in Python 3 (statsmodels, SciPy, pingouin, and NumPy). Cross-sectional end points were analyzed by two-way ANOVA (conditioning × treatment) using Type III sums of squares with sum-to-zero (deviation) coding. Effect sizes are reported as partial eta-squared (η*p* ^2^) with 90% confidence intervals derived from the noncentral *F* distribution and, for pairwise contrasts, as Hedges’ *g* with 95% bias-corrected bootstrap confidence intervals (5000 resamples). As a distribution-free corroboration, each factorial effect was retested by aligned rank transform (ART) ANOVA.^43^ The stagewise progression of V̇O_2_ during the incremental test was modeled with a linear mixed-effects model, including a random intercept per animal; stage was treated as a categorical factor, and this specification was compared against a linear stage term by the Akaike information criterion and a likelihood-ratio test, with the interaction confirmed by a parametric bootstrap likelihood-ratio test. Within predefined families of related end points, *p*-values were adjusted for multiplicity by the Benjamini–Hochberg (false discovery rate) and Holm (family-wise error rate) procedures. The family for the treatment effect comprised the five end points measured in the full cohort—body mass, open-field distance, absolute V̇O_2_max, mass-normalized V̇O_2_max, and running power; blood lactate, measured in a subset of animals, and the cardiac stereological end points were treated as separate families. All tests were two-sided with α = 0.05. Because the cardiac interpretation rests on null results, the treatment and interaction effects on cardiomyocyte Vv were additionally bounded by 90% confidence intervals, reported as the smallest effects the design excludes; no equivalence margin was prespecified, so these bounds are descriptive and are interpreted relative to the training effect estimated in the same model. Data are summarized as mean ± standard deviation in the text and as mean ± standard error of the mean in the figures. The number of biological replicates was n = 8 per group for the ergospirometric and open-field end points (32 animals), n = 6 per group for the rotarod (24 animals), and n = 6–7 per group for cardiac stereology (25 animals).

## Data Availability

The data underlying this study—raw and processed measurements of aerobic capacity and cardiac stereology, together with a data dictionary and study documentation—are openly available in the Open Science Framework at 10.17605/OSF.IO/EJDNG (CC BY 4.0). The analysis code that reproduces all reported statistics is openly available at 10.17605/OSF.IO/6258S (MIT License).
